# Leg movements affect speech intensity

**DOI:** 10.1152/jn.00282.2022

**Published:** 2022-09-21

**Authors:** Hélène Serré, Marion Dohen, Susanne Fuchs, Silvain Gerber, Amélie Rochet-Capellan

**Affiliations:** ^1^GIPSA-lab, CNRS, Grenoble Institute of Technology, University of Grenoble Alpes, Grenoble, France; ^2^Leibniz-Centre General Linguistics (ZAS), Berlin, Germany

**Keywords:** acceleration peaks, amplitude envelope, fundamental frequency, limb-speech interaction, physical impulse

## Abstract

Coordination between speech acoustics and manual gestures has been conceived as “not biologically mandated” (McClave E. *J Psycholinguist Res* 27(1): 69–89, 1998). However, recent work suggests a biomechanical entanglement between the upper limbs and the respiratory-vocal system (Pouw W, de Jonge-Hoekstra D, Harrison SJ, Paxton A, Dixon JA. *Ann NY Acad Sci* 1491(1): 89–105, 2021). Pouw et al. found that for movements with a high physical impulse, speech acoustics co-occur with the physical impulses of upper limb movements. They interpret this result in terms of biomechanical coupling between arm motion and speech via the breathing system. This coupling could support the synchrony observed between speech prosody and arm gestures during communication. The present study investigates whether the effect of physical impulse on speech acoustics can be extended to leg motion, assumed to be controlled independently from oral communication. The study involved 25 native speakers of German who recalled short stories while biking with their arms or their legs. These conditions were compared with a static condition in which participants could not move their arms. Our analyses are similar to that of Pouw et al. (Pouw W, de Jonge-Hoekstra D, Harrison SJ, Paxton A, Dixon JA. *Ann NY Acad Sci* 1491(1): 89–105, 2021). Results reveal that the presence of intensity peaks in the acoustic signal co-occur with the time of peak acceleration of legs’ biking movements. However, this was not observed when biking with the arms, which corresponded to lower acceleration peaks. In contrast to intensity, F0 was not affected in the arm and leg conditions. These results suggest that *1*) the biomechanical entanglements between the respiratory-vocal system and the lower limbs may also impact speech; *2*) the physical impulse may have to reach a threshold to impact speech acoustics.

**NEW & NOTEWORTHY** The link between speech and limb motion is an interdisciplinary challenge and a core issue in motor control and language research. Our research aims to disentangle the potential biomechanical links between lower limbs and the speech apparatus, by investigating the effect of leg movements on speech acoustics.

## Graphical abstract

**Figure:**
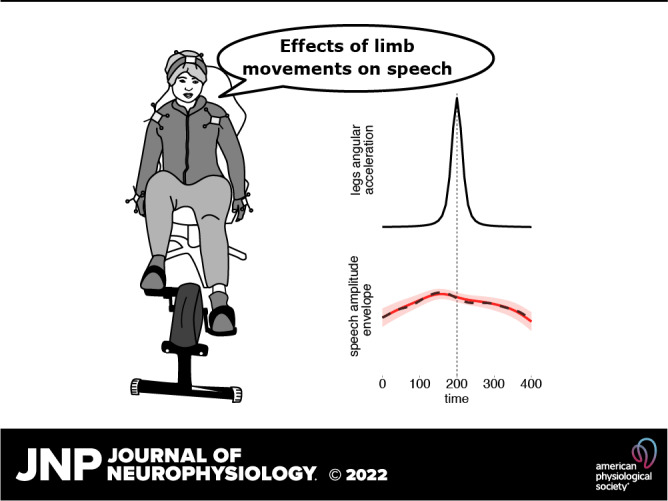

## INTRODUCTION

In everyday life, speech is most often produced while moving the limbs for different purposes. This is one of the major arguments in favor of the oral modality over the manual modality for human language in evolution: it is possible to speak while performing a large range of related or unrelated actions with our arms and hands. Contrastively, spoken language studies in laboratory situations are most often conducted in motionless conditions. The current work investigates the effect of limb (hand or leg) motion (versus immobility) on spontaneous speech acoustics, focusing on fundamental frequency and intensity.

This study is grounded on the following assumption: since limb and speech movements frequently co-occur, and rapid limb motions influence speech acoustics, the brain may have adapted to this co-occurrence and controlled them jointly during learning instead of using additional effort to counteract them. This control may rely on biomechanical patterns, i.e., high acceleration of limb movements increases the pressure exerted on the lungs. Higher subglottal pressure causes speech intensity to rise and an increase in fundamental frequency. In motor control, it is known that the nervous system uses predictions about biomechanical properties specific to the context to plan a movement (1) and to minimize metabolic energy cost through sensorimotor processes (2). In the context of our study, this would mean that speech motor control and leg movement control are synchronized to minimize effort cost. Even further, the nervous system is minimizing energy expenditure by considering biomechanical properties. It is possible that the effect of leg movements on speech acoustics via respiration is one of the parameters considered by the nervous system, and the best way to minimize effort cost is to place stressed syllables on acceleration peaks of limb movements.

Previous studies along those lines have discussed that one of the systems playing a crucial role in allocating energy resources between speech and limb movements is breathing (3). Breathing adapts flexibly to the production of speech (4) as well as to limb movements (5). When speech and limb movements co-occur, breathing becomes a shared resource shaped by the constraints of the two systems. Hence, breathing is increasingly studied as a central aspect of the relationship between speech and a range of limb movements such as manual gestures accompanying speech in communication (3, 6–8); arm movements for object manipulation (9, 10); or leg movements for locomotion (11, 12).

Recently, Pouw et al. (3, 6, 7, 13) suggested a biomechanical link between upper limb movements and speech acoustics in relation to anatomical constraints between the arms and the breathing system. They indicated that abrupt accelerations or decelerations of the upper limbs have a cascading effect on the respiratory system. They increase subglottal pressure, which may have consequences on acoustic parameters of speech. In particular, the amplitude envelope and fundamental frequency (F0) increase close to the deceleration peak of the arm movement. These effects were also measured for a vertical rhythmic flexion-extension movement of the wrist, but to a lesser extent. The latter observations were made for syllable production (3, 6) and spontaneous speech (13). Pouw et al. interpreted their results in line with a phylogenetic account: when humans became bipedal, not only was their respiratory system freed from biomechanical constraints due to locomotion, but the upper limbs were also freed and may have modulated the respiratory-vocal system, which was less skilled at that time. In this way, the upper limbs may have contributed to refining respiratory control when speaking.

Aside from this potential biomechanical effect, there is growing evidence that gestures and speech are tightly coupled at a motor level (14–17). This coupling is reflected in prosody (18, 19) and emphatic stress (20, 21). Studies of deixis provide a set of arguments for the interplay between arms and speech. Previous work suggested an anchoring of spoken language in prosodic deixis. As an illustration, prominent syllables have been shown to occur close to the apex of a pointing gesture (22, 23) and prosodic focus attracts this same apex (24, 25). Depending on the prosody of a language, speakers may learn specific coordination between the speech and gesture motor systems so that prominent syllables are tightly synchronized with the gesture target. Breathing may play an essential role in this synchronization process as the breath group has long been recognized as an important unit of prosody (26).

Much less work has been carried out for lower limb movements. However, they also impact breathing (27, 28) due to oxygen demands and anatomical constraints. In particular, the abdominal viscera move up and down (on the longitudinal axis) during jumping or walking, acting as a piston on the diaphragm. This piston effect can be caused by a spinal flexion: the trunk is shortened by flexion of the lumbar, moving up the viscera and the diaphragm. Finally, pectoral, intercostal, and abdominal muscles are used for locomotion and breathing. Their contraction may trigger expiratory flow and could be reflected in speech parameters sensitive to airflow, such as amplitude envelope and F0.

Exercise involving the lower limbs affects speech acoustics: average speech intensity and average F0 increase while biking at different physical effort levels (11, 12, 29). F0 also increases with effort during or after a treadmill task (30, 31). Trouvain and Truong (31) suggested that this increase comes from a higher subglottal pressure during physical effort. However, other studies highlight inter-speaker variability. Participants have different and sometimes even opposite behaviors regarding the evolution of their F0 while performing limb movements: some increase F0, some decrease it, and for others, F0 remains unchanged (12, 32, 33). Besides inter-speaker variability, the relationship between exertion level and F0 is not linear: Johannes et al. (34) found that F0 is impacted by exercise only close to exhaustion. There may also be local effects of motion on F0 that might not appear when F0 is averaged over a trial. The local effect of motion on F0 may depend on the peak acceleration value of the movement (3, 6).

To further address the biomechanical link between limb motions and speech, the present study investigates the effect of limb movements on acoustic parameters of spontaneous speech during biking. Intensity and fundamental frequency of narrative speech are analyzed during biking motions with the arms or with the legs compared with a control condition with no motion. Biking movements were used as they are relatively automatic and do not require a large attention span, spatial navigation, or visual control. Moreover, they can be performed with the legs or arms, and the movement amplitude is wholly constrained. As physical effort could strongly affect breathing due to oxygen needs, biking movements were performed at a comfortable rhythm for the participant. Furthermore, arm or leg biking motions may induce the contraction of muscles involved in breathing or antagonist muscles impacting the thoracic cage. We aimed to test whether these motions, with low energy demands and no synchrony constraints, generate F0 and intensity peaks in speech at the highest physical impulse of the motion (the acceleration peak within each movement cycle). If so, this would be supplementary evidence in favor of a biomechanical influence of limb movements on speech via breathing. The data were analyzed using an approach similar to Pouw et al. (13), investigating the nonlinearity in the speech amplitude envelope and F0 around the acceleration peak in limb movements. Investigating nonlinearity in the parameters of acoustic parameters is relevant for testing whether there is a local effect of the physical impulse on acoustics, resulting in a “bump” in the acoustic time course within 400 ms around the acceleration peaks. We first looked at linear effects to investigate the correlation between the values of the acceleration peaks and the acoustic values. The idea was to see if the acoustic values (F0 and envelope) around the physical impulse increase with the value of the acceleration peak.

## EXPERIMENT

The effect of limb acceleration on speech acoustics was investigated based on the recording of speech, breathing, and limb motion data during a narrative task in different conditions of limb movements (see Ref. 8, for more details). The data were recorded at the Leibniz-Zentrum Allgemeine Sprachwissenschaft (ZAS). The procedure was approved by the Ethical Board of the German Linguistic Society.

### Participants

Twenty-five healthy participants took part in this experiment. Because of technical issues, there was a problem with saving the movement signals for three participants. Hence, we ran the analysis on 22 participants (16 females and 6 males) aged 20–29 yr [mean = 23.5 yr; standard deviation (SD) = 2.6 yr]. They were registered on the participant database Lingex and replied to an email announcement. All participants spoke German as their mother tongue and reported no respiratory, motor, speech, neurological, or auditory impairments. They signed an informed consent form and received 10€ per hour for their participation.

### Material for the Narrative Task

Four animation movies were created for this experiment. Each of them depicts the story of an alien arriving on Earth with pictures and a narrator telling the story. The structure of the story was based on the work of Mandler and Johnson (35). Each story consists of 264–300 words, with the same number of images. As an example, one movie is available on the OSF repository; https://doi.org/10.17605/OSF.IO/DETHU.

### Experimental Setup and Data Acquisition

Participants sat on a chair during the whole session. Their speech was recorded using a stand-up microphone (MKH 50 P48, Sennheiser) placed at 50 cm from their mouth at a 44,100 Hz sampling frequency. Participants’ movements were recorded using motion capture (OptiTrack, Natural-Point Inc., cameras prime 13 recording at 200 Hz) and video recordings. The motion capture markers were assembled as rigid bodies (for more reliability in the detection) and placed on the head, the back, the shoulders, and the hands of the participants. A minibike (SportPlus, Hamburg, Germany) was placed either on a table in front of the participant when biking with the hands, or at her/his feet when biking with the legs. A marker was attached to each pedal.

Only the motion capture data of the markers on the pedals and the acoustic speech signal are analyzed in this paper. Note that breathing was synchronously monitored using an Inductotrace inductance plethysmography system (see Ref. 8). However, we did not analyze breathing for the current purpose: since the time window analyzed is around 400 ms around the physical impulse, it is impossible to know whether the potential bumps we could find in breathing kinematics at the time of occurrence of the physical impulses come from a shift of the belts due to the cycling movement or from a real effect of the impulse on the thoracic cage.

### Task

On *days 1* and *2*, the participants’ main task was to watch each story and then recall it in one of the following conditions: *1*) arms free: speaking without any constraint on limb movements (armFree); *2*) arms blocked: speaking with the hands holding the chair (armBlock); *3*) biking with the legs: speaking while comfortably biking with the legs (legMot); and *4*) biking with the arms: speaking while comfortably biking with the arms (armMot).

To ensure equal balance between order of presentation of the stories, order of the conditions, and condition-story associations, we used pseudo-randomization (see Ref. 8). With 24 possibilities for condition orders and 24 potential story-condition associations, 576 participants would have been required for complete randomization which was not feasible. We chose to record 24 participants in order for each story to be presented six times in each condition and position. Unfortunately, one participant did not come back on the second day. She was replaced by another who also failed to come back on *day 2*. We however decided to keep the data of these two participants even though it slightly unbalanced the number of times a given story appeared in a given condition (6 or 7 times) and the number of times a condition was presented at a given position (5 to 8 times).

### Procedure

On *day 1*, participants completed forms and performed the baseline for the breathing recordings. They then performed the narrative task (watching and listening to the story and recalling it) in the four experimental conditions.

On the following day (*day 2*), they freely recalled everything they could remember about the stories in the armFree condition and then did the main task again in the same conditions as on *day 1*. Participants were finally invited to return 10 days later to assess long-term memory of the stories without motion. These data are not relevant for the current analysis as they did not involve biking motion.

## DATA ANALYSES

We analyzed the motion capture signals from the markers on the pedals of the bike, and the speech acoustic data in the conditions arms blocked (armBlock), leg motion (legMot), and arm motion (armMot). The condition armFree was not included due to the large interpersonal variability in the use or lack of co-speech gestures. Moreover, speech and gesture coupling in spontaneous speech is strongly related to communicative and cognitive factors.

### Data Processing

#### Processing of biking movements.

The time series of the positional coordinates of the motion capture markers were extracted in a csv file format. The marker of the right pedal was detected using a code in Python to parse the file and locate the markers moving circularly. The coordinates of the vertical position were saved in a new csv-file at 200 Hz. The other dimensions were not used because the bike experienced some small displacements, especially on the horizontal plane, through the trials.

**Figure 1.**
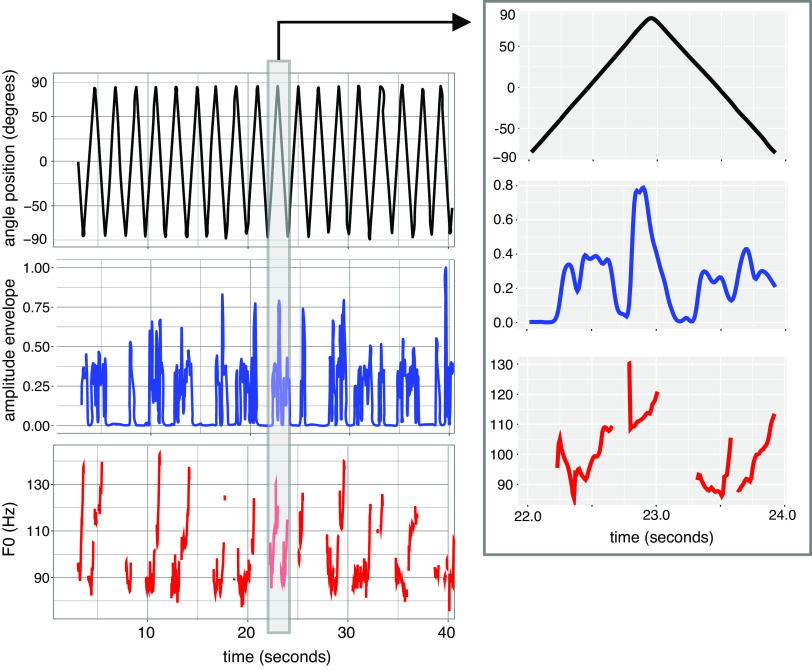
Example of analyzed signals for one participant in the legMot condition. *Left*: sample of biking angular trajectory (*top*) and corresponding parameters in the acoustic signal: amplitude envelope (*middle*) and F0 (*bottom*). *Right*: zoom in on one biking cycle for each parameter.

The extracted coordinate vector was resampled at 100 Hz and filtered with a Butterworth filter at a cutoff frequency of 10 Hz. The signal peaks (one peak for each cycle) were detected using the peak finder function of MATLAB (36) with adequate parameters validated for each participant based on signal visualization. Results of the detection were visually inspected. Each cycle was characterized by its duration (from 0.68 s to 2.9 s, mean = 1.2 s, SD = 0.37 s). Each session started with 2 s of no movement during which the breathing recording was launched, and each participant needed one to two biking cycles to reach their comfortable rhythm before starting to speak. We decided to remove this part by discarding the coordinates before the first complete biking cycle. The signal was transformed with the arc sinus function to express displacement as an angle of rotation (cf. Fig. 1, *row 1*). As can be seen in Fig. 1, the signal is a few degrees below 180° because of tiny fluctuations around the peaks of the vertical position signal (also see Supplemental Fig. S1; see https://doi.org/10.17605/OSF.IO/TSYJK). The acceleration was computed as the second derivative of the position signal.

#### Speech parameters.

The fundamental frequency (F0) was extracted from each speech signal using the Praat software (37; autocorrelation method, range = 50–250 Hz for males and 100–300 Hz for females; cf. Fig. 1, *row 3*). The values above the third quartile plus three times the inter-quartile space and below the first quartile minus one time the inter-quartile space were discarded. These thresholds were chosen after looking deeply into the data and noticing that most of the values between the third quartile plus one time and three times the interquartile range were not outliers. On average 3% of all the data was discarded.

After downsampling the speech signal to 11,025 Hz, the amplitude envelope was extracted and processed using the method of Pouw et al. (13): the envelope function of MATLAB (36), using a Hilbert transformation, was applied to the speech signal. The upper bound of the output was filtered using a 5-Hz Hanning window, and the signal was smoothed using a Gaussian-weighted moving average over each window of 1,000 points (see Fig. 1, *row 2*). The scripts are available on the OSF repository; https://doi.org/10.17605/OSF.IO/ZFV75.

To temporally align the values of the acoustical parameters (F0 and intensity) with the movement signals, all were re-sampled to 100 values per second (100 Hz).

After preprocessing, the movement and synchronous acoustic signals were paired for legMot and armMot conditions. As there was no biking signal in the armBlock condition, a surrogate condition was created. For each speech_armBlock trial, a biking signal was randomly chosen from the legMot or armMot condition of another participant. The longest signal was then cut to align with the shortest one.

Each biking cycle encompasses two acceleration peaks, corresponding to the effort on the right pedal and the effort on the left pedal. We considered the acceleration peaks of both pedals. To extract them, we used the signal of the right pedal only: since the bike is a rigid body, the acceleration applied on the left pedal was fully reflected on the right pedal.

Since the goal of the study is to investigate the impact of motion on F0 and intensity, only cycles with speech, but not within a pause were extracted. To do so, a native speaker of German annotated all the files in Praat (37) to label the interpausal unit intervals, i.e., uninterrupted speech units without silent pauses. Only these intervals were kept in the analyses.

### Statistical Analyses

The scripts and the data sets of the statistical analyses are available in the repositories; https://doi.org/10.17605/OSF.IO/K8J6T, https://doi.org/10.17605/OSF.IO/XHK25, https://doi.org/10.17605/OSF.IO/8S5BU.

#### Predicting the magnitude of F0 and amplitude envelope peaks based on the magnitude of acceleration peaks.

To investigate the effect of limb movements on speech, we first evaluated if the amplitude of the acceleration peaks in the biking data could predict the amplitude of the acoustic peaks in the speech signal (F0 and amplitude envelope) calculated as the average value 50 ms around the time of occurrence of the acceleration peak (*T*_accPeak_).

A linear mixed model was applied to F0. The predictors are the acceleration magnitude 50 ms around *T*_accPeak_, day, condition, and their interactions. Participant was included in the model as a random effect with random slopes on the condition factor, and the residual variability was corrected on the day, meaning that the distribution of the residuals was corrected so that it was the same on *days 1* and *2*.

For the analysis of intensity, the amplitude envelope was z-scaled per trial. The differences in the overall level of the amplitude envelope between conditions were an artifact resulting from variations in the mouth-microphone distance. Since the participant was moving her trunk forward to reach the bike when biking with the arms, the mouth-microphone distance was changed, and so was the intensity. Because of this z-scaling, the trials in the different conditions and days were no longer comparable, so the only predictor for the envelope is the acceleration magnitude 50 ms around *T*_accPeak_. Participant was included in the model as a random effect with a random intercept.

#### Changes in acoustic parameters related to motion acceleration.

We also evaluated a possible effect of biking acceleration on F0 and on the amplitude envelope by assessing their nonlinearity around the limb acceleration peaks (*T*_accPeak_) (13).

Non-linearity in the armMot and legMot conditions was compared with that of the surrogate armBlock condition. A generalized additive mixed model (GAMM) was applied to the F0 and amplitude envelope time series using a time window of 200 ms before and after the acceleration peak of each biking cycle (400 ms in total). The GAMM uses basis functions, called smooth, to estimate the shape of the curve over time. The time series of F0 and amplitude envelope were averaged by participant, condition, and day to follow the method of Pouw et al. (13) and avoid high intraspeaker variability due to spontaneous speech. This variability was also compromising the goodness of the fit of the model and the compliance with the model assumptions.

To test whether the biking conditions are nonlinear and different from the surrogate condition, a smooth was set per condition for both F0 and amplitude envelope. For the F0 analysis, sex, condition, and day were also added as fixed factors. After a backward selection, all the factors remained in the model. Because of the artifact resulting from variations in the mouth-microphone distance, amplitude envelope was *z*-scaled per trial. Because of this transformation, the trials in the different conditions and days were no longer comparable. Thus, we did not add sex, condition, and day for predicting the amplitude envelope, and the analysis thus differed from the F0 model.

For both F0 and envelope, the participant variable was included as a nonlinear random effect with a random smooth for participant and condition. Since this analysis focuses on F0 and amplitude envelope as time-series, the correlation from one point of the time-series to the next has to be considered: the value at a given time *t* is dependent on the value at the time *t* − 1, and possibly before. A term to consider the potential autocorrelation between the consecutive points within the time series was therefore included. We ran the analysis using the R software (38) environment and the mgcv package (39), following Martijn Wieling’s tutorial (40), as well as the nlme package (41). The nonlinearity of the curves and the difference between the curves across the conditions were tested in two different models (see Ref. 40).

For the amplitude envelope and F0, we expected the acoustic curves to be nonlinear in the biking conditions and different from those in the armBlock condition.

## RESULTS

### General Description of the Data Set

All the effects and differences described in the RESULTS section were confirmed with statistical analyses. Details of these analyses are available in the Supplemental Material.

#### Biking cycles.

The distribution of the number of cycles per participant and per day is described in Table 1. Neither the day nor the condition had an effect on this distribution.

**Table 1.** Total number of biking cycles and number of biking cycles occurring within an interpausal unit per participant, day, and biking condition

	All Cycles			Cycles Occurring Inside an IPU		
Conditions	Minimum	Median	Maximum	Minimum	Median	Maximum
armBlock *day 1*	42	80	196	20	46	110
armBlock *day 2*	19	93	171	10	52	123
legMot *day 1*	29	93	222	14	56	138
legMot *day 2*	36	85	248	15	70	159
armMot *day 1*	24	86	158	10	44	81
armMot *day 2*	31	84	171	19	49	103

IPU, interpausal unit.

Considering the average duration of the cycles in general, leg movements were significantly shorter (mean = 1.07 s, SD = 0.29) than arm movements (mean = 1.3 s, SD = 0.37), and arm movements were 0.3 s shorter on *day 2* than on *day 1* (no effect of day for leg movements). After averaging the durations of the arm and leg movements per participant and day, the correlation between arm and leg movement durations was weak but significant (*r*^2^ = 0.3, *t* = 4.16, df = 41, *P* = 0.00016).

#### Interpausal units.

The median value of the duration of the interpausal units in speech signal was 3.4 s (mean = 3.65 s, SD = 2.28), with the first quartile at 1.8 s, and the third quartile at 5.2 s. The distribution of interpausal unit durations is similar in all the conditions. On average, the duration of the interpausal units increased by 0.45 s from *day 1* to *day 2*.

### Predicting the Magnitude of F0 and Amplitude Envelope Peaks Based on the Magnitude of Biking Movement Acceleration Peaks

The results of the models predicting the peak values in F0 and amplitude envelope from the peak values in motion acceleration are given in Table 2.

**Table 2.** Parametric coefficients of the linear mixed model with F0 (averaged over 50 ms around each T_accPeak_ and normalized per participant) as the dependent variable and day as a predictor

	Value	Std. Error	DF	*t* Value	*P* Value
Env (z scaled)
Intercept	−0.00001	0.008	12,707	−0.001	1
Acceleration	0.002	0.003	12,707	0.73	0.4
F0
Intercept	185.2	10.17	9,316	6.46	<1*e*-6
Acceleration	0.05	0.08	9,316	0.47	0.56
* Day 2*	−4.25	0.54	9,316	−7.90	<1*e*-6

F0 values around *T*_accPeak_ were predicted by the peak values of the acceleration, the day, the condition, and their interactions. After a backward selection, only the day remained in the model and had an effect on F0 (*b* = −4.25, *z* = −7.90, *P* < 1*e*-6).

As for the prediction of the amplitude envelope magnitude around *T*_accPeak_, the only predictor was the acceleration, and it did not significantly affect the amplitude envelope values.

**Figure 2.**
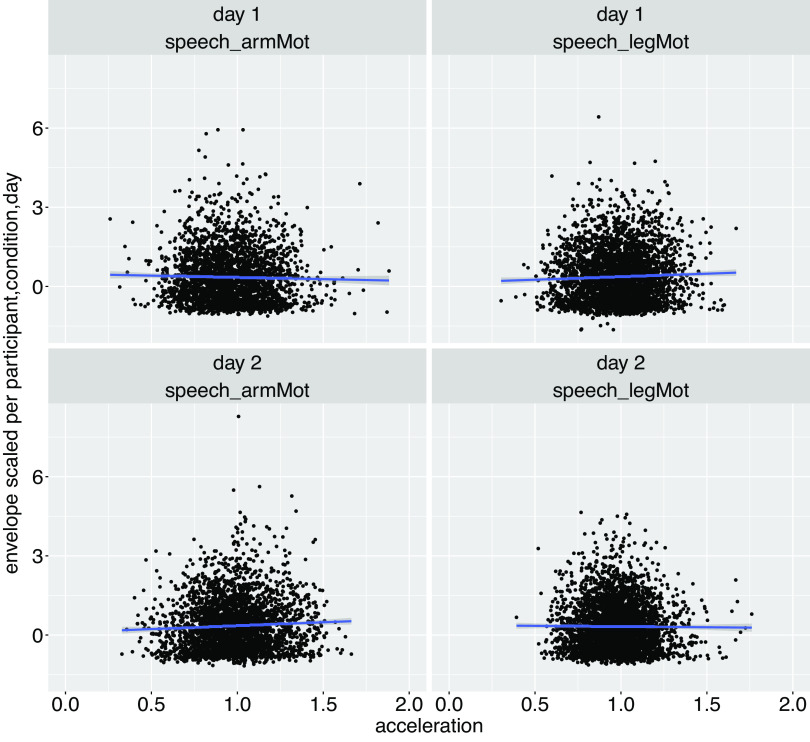
Amplitude envelope values (averaged over 50 ms around each *T*_accPeak_ and normalized per participant, day, and condition) plotted against acceleration peak values. Blue line: estimate of the linear relationship between the acceleration peak values and amplitude envelope values [estimated with the function geom_smooth R (38)].

**Figure 3.**
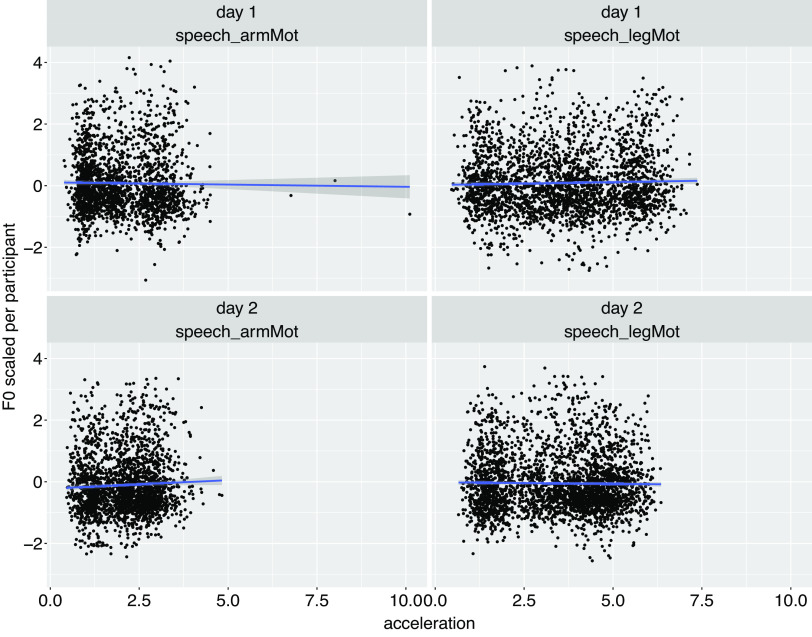
F0 values (averaged over 50 ms around each *T*_accPeak_ and normalized per participant) plotted against acceleration peak values. Blue line: estimate of the linear relationship between the acceleration peak values and F0 values [estimated with the function geom_smooth R (38)].

No significant correlation was observed between the values of the motion acceleration peak and the values of F0 or amplitude envelope (averaged 50 ms around *T*_accPeak_) (see Figs. 2 and 3).

### Changes in Acoustic Parameters Related to Motion Acceleration

**Figure 4.**
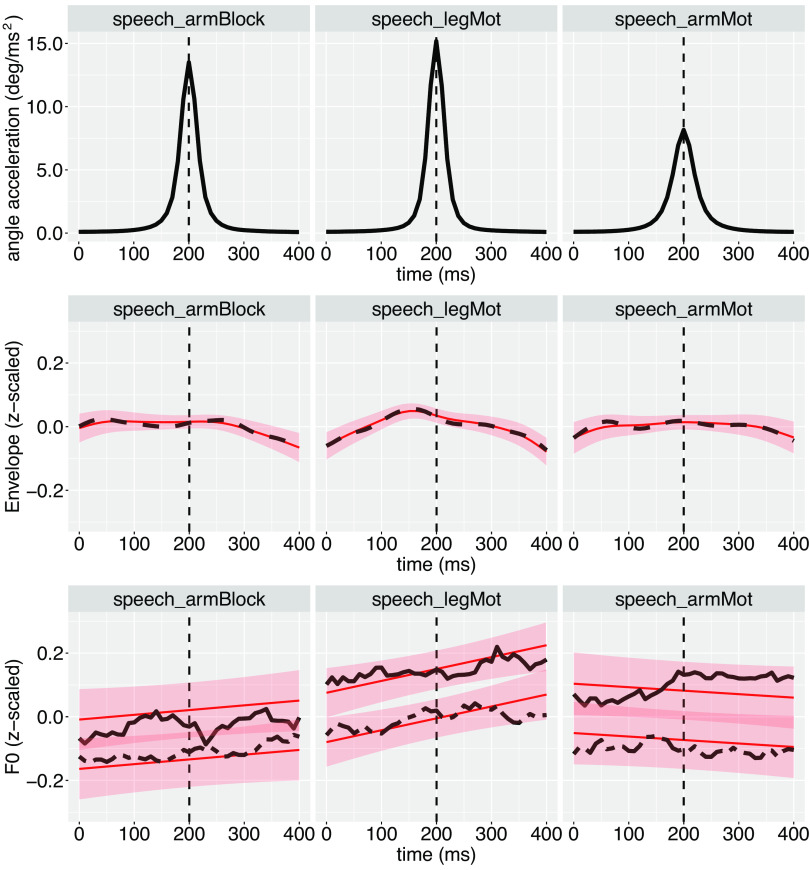
Average acceleration of the biking movement (*top*), amplitude envelope *(middle*), and F0 (*bottom*) of the speech signal 200 ms before and after the biking acceleration peak. Red: smooth estimates of the evolution of the acoustic parameters over time, per condition. The shadow part represents the 95% confidence interval. The vertical dashed lines represent the time of occurrence of the biking acceleration peak. For F0 (third line), the solid line on F0 represents the first day. The irregular dashed line represents the second day.

The effect of the condition on the time series of the acoustic parameters around the movement acceleration peak is illustrated in Fig. 4.

Table 3 summarizes the overall effects of the factors sex, day, and condition on F0. The overall level of F0 was higher for females than for males (*b* = 97.39, *z* = 19.11, *P* < 0.0001). As can be seen in Fig. 4, it was also lower on *day 2* compared with *day 1* (*b* = 3.8, *z* = 14.67, *P* < 0.0001). The overall level of F0 did not increase significantly when biking with the legs or with the arms as compared with when the hands were blocked (see Table 3 and Fig. 4).

**Table 3.** Parametric coefficients of the intercepts of the GAMM for F0

	Contrast	Estimate	Std. Error	*t* Value	*P* Value
F0	Female-male	97.39	5.09	19.11	<2*e*-16***
	*Day 1*-*day 2*	3.8	0.26	14.67	<2*e*-16***
	armBlock-legMot	1.71	5.54	0.31	0.76
	armBlock-armMot	3.43	5.5	0.62	0.53

***P < 0.001. GAMM, generalized additive mixed model.

The output of the generalized additive mixed models testing for nonlinearity is given in Table 4 and the one for differences between the conditions in Table 5.

**Table 4.** Approximate significance of the nonlinearity of the smooth terms

	edf	Red.df	*F*	*P*
Env (z scaled)
Smooth_armBlock	4.47	5.01	3.17	0.007**
Smooth_armMot	4.55	5.07	2.15	0.05.
Smooth_legMot	6.05	6.63	7.77	<2*e*-16***
F0
Smooth_armBlock	1.00	1.00	1.28	0.26
Smooth_armMot	1.00	1.00	1.45	0.23
Smooth_legMot	1.00	1.00	5.5	0.02*

“edf” can be seen as an estimate of the number of parameters needed to compute the smooth. “Red.df” is the number of degrees of freedom for testing the hypothesis. *P < 0.05, **P < 0.01, ***P < 0.001.

**Table 5.** Approximate significance of the difference between the smooth terms in the different conditions

	edf	Red.df	*F*	*P*
Env (z-scaled)
S(legMot-armBlock)	5.22	5.76	2.35	0.03*
S(armMot-armBlock)	1.00	1.00	1.15	0.28
F0
S(legMot-armBlock)	1.00	1.00	0.68	0.41
S(armMot-armBlock)	1.00	1.00	2.27	0.13

“edf” can be seen as an estimate of the number of parameters needed to compute the smooth. “Red.df” is the number of degrees of freedom for testing the hypothesis. *P <0.05.

For F0, the only significant nonlinearity was when biking with the legs (edf = 1, *F* = 5.5, *P* = 0.02; see Table 4). The curves in the legMot and armBlock conditions are not significantly different. The same is true for the armMot and armBlock conditions (see Table 5).

For the amplitude envelope, the curves are significantly nonlinear over time in the armBlock (edf = 4,47, *F* = 3,17, *P* = 0.007; see Table 4) and legMot conditions (edf = 6.05, *F* = 7.77, *P* < 0.0001), with a tendency to be nonlinear in the armMot condition (edf = 4.55, *F* = 2.15, *P* = 0.05). The curve in the legMot condition is significantly different from the curve in the armBlock condition (edf = 5.22, *F* = 2.35, *P* = 0.03; see Table 5).

## DISCUSSION

In previous work, Pouw and coworkers (6, 7, 13) suggested a potential biomechanical effect of upper limb movements on F0 and speech amplitude envelope. In their studies, participants produced relatively abrupt arm movements leading to high acceleration peaks. These acceleration peaks correlated positively with speech acoustics, specifically with peaks in the amplitude envelope and F0. The authors also showed that the acoustic events occurred in the vicinity of the acceleration peak. Although the original work was based on controlled speech units (6, 7), the relationship has been recently confirmed with natural speech (13) and partially in Indian vocal music (42). In the current study, we extended this work to cyclical movements of the arms or the legs with low physical effort during spontaneous narration.

In contrast to Pouw et al. (13), in our study, participants produced biking movements, either with the arms or with the legs. The contribution of our study lies in considering smoother movements, with other limbs than the arms. Rhythmic motion activities are frequent in everyday life, during which people can talk to each other, e.g., walking and talking on the phone.

Our results did neither show a correlation between the acceleration peaks during biking motion and fundamental frequency peaks, nor a correlation with the speech amplitude envelope. We also investigated the potential nonlinearities of the acoustic parameters around the acceleration peaks. For F0, the data did not show an effect in any of the biking conditions. For the amplitude envelope, however, acceleration peaks yielded a significant nonlinear effect when biking with the legs but not with the arms.

We interpret the absence of an effect in line with a threshold of movement acceleration that is needed to induce the cascading mechanisms of gesture-speech physics. In the study by Pouw et al. (13), acceleration peaks were higher than 4 cm/s^2^ with one maximal value at 16 cm/s^2^. In our study, acceleration values for arm motion ranged from 0.4 to 10 cm/s^2^ with a median value of 2 cm/s^2^ whereas for leg motion it ranged from 0.5 to 7.4 cm/s^2^, with a higher median value of 3.5 cm/s^2^ relative to the arms. When participants biked with their legs, they produced higher acceleration peaks than when they biked with their arms. This is congruent with the fact that legs have larger muscles, they are heavier, and have an advantage for endurance tasks such as biking (43).

Although we did not find any linear relationship between acoustic peaks and physical impulse, we did find a nonlinear effect of the leg acceleration peaks on the time course of intensity. It means that the legs’ physical impulse promptly increases intensity, but the magnitude of this increase does not depend on the magnitude of the acceleration. Again, this result can be due to the range of values of acceleration. This range may not be wide enough, with higher values, to see a linear effect.

The absence of an effect on fundamental frequency might be multifaceted. In the study by Pouw et al. (13), gesture-speech physics was present in F0, but the relation was weaker than the one with intensity. It might be possible that even a higher acceleration peak is needed to trigger an increase in F0 (see Ref. 34). The relation between subglottal pressure, F0, and intensity has also been debated (for an introduction see Ref. 44). At the temporal scale of approximately a second, Lieberman (26) suggested that the overall decrease in F0 over the course of a sentence would be a consequence of decreasing subglottal pressure. In contrast, Ohala (45) suggested that fundamental frequency can flexibly change depending on the intended communicative goals. He argued that the control of the larynx must be quick, flexible, and independent because its primary function in phylogeny is lifesaving, i.e., to protect the lungs from external objects. Hence, if motion is not abrupt enough, phonation may not be perturbed due to gesture-speech physics. Other studies have shown that abrupt pushes on the upper chest led to higher subglottal pressure and higher F0 values in sustained phonation due to laryngeal reflexes (46). As a secondary function, F0 plays an essential role for segmental (in tone languages) and prosodic characteristics in a diverse set of languages. It also varies with respect to communicative constraints. At the level of short temporal windows (e.g., a word under focus), local subglottal pressure changes have been found to correlate positively with changes in intensity but not in F0 (44). Besides, biking movements are continuous, cyclical, and predictable with no clear target or turning point. Due to the predictability of the movements, the effect of motion on F0 could be counteracted, e.g., by changing the vertical position of the larynx or the stiffness of the vocal folds. The continuous and cyclical nature of the produced motion with no changes in amplitude may further be responsible for the negative finding.

Several studies in the literature report a tight coordination between pointing or beat gestures and F0 targets (47, 48). It is also possible that since biking movements are not likely to have a communicative function, this upper limb motion does not impact speech acoustics. However, when biking with the legs, speech intensity does increase when acceleration peaks occur. Since leg movements are not likely to be communicative, especially biking, this result suggests that this effect comes from biomechanical processes: pectoral, intercostal, and abdominal muscles may function for biking and speaking. Their contraction during physical effort may trigger expiratory flow, which increases speech intensity. This hypothesis is in line with previous findings in the literature (11, 12).

In everyday life, speech often takes place together with body motion. These movements can be linked to speech and help the speaker reach communicative targets (21). They can also be independent or even competing with speech by constraining the hands, such as washing dishes while talking with someone or biking with a friend. Suppose the physical effort implied requires the contraction of muscles also used in active breathing, such as during an acceleration or deceleration peak. In that case, the body has to absorb this physical impulse to avoid an impact on speech. For F0, this impact may be counteracted by the larynx up to a certain level of impulse, but for the amplitude envelope, such compensation mechanisms may not take place, making speech intensity more sensitive to body movements. On the other hand, these biomechanical constraints may have been learned by the sensorimotor system and may be used as a tool to optimize the coupling between speech and communicative movements. For instance, beat gestures may be synchronized with emphatic stress because their physical impulse facilitates the increase of speech intensity (21).

In summary, consistent with previous work (13, 44), our results suggest that intensity seems to be more sensitive to the biomechanical impact of light limb movements than F0. However, our findings do not consistently show an effect of motion on F0. As mentioned earlier, the physical impulse involved in biking with the arms in our study may not be high enough to trigger a trunk tensioning that could have an impact on speech acoustics. Further work should investigate the threshold for motion acceleration value to induce an effect on speech intensity and F0. This threshold might be speaker-specific. Our study brings insights into how biomechanical processes can support the synchronization between limb movements and speech. Based on our analyses, we suggest: *1*) this link starts at a certain threshold but is not visible below; *2*) the link is more present in intensity than in F0, and *3*) not only do upper limb movements have an effect on acoustic parameters, but also movements of other body parts, such as the legs.

The relevance of studying this biomechanical process lies in the way it can influence motor control of co-occurrent speech and limb movements: to plan a movement, the nervous system uses predictions about biomechanical properties specific to the context (1) and to minimize metabolic energy cost through sensorimotor processes (2). The biomechanical effect at work between limb movements and speech is likely to influence the nervous system to optimize energy expenditure: during a periodic movement such as biking, the increased lung pressure at acceleration peaks is predictable. Either speech or limb control can adapt to enable the occurrence of both stressed syllables and physical impulses at the same time. This potential phenomenon may be more visible at higher biking rates, when the optimization of energy expenditure is necessary to allocate breathing resources between speech and movements, and limit fatigue. On the other hand, as mentioned in our introduction, gestures and speech are tightly coupled at a motor level. This coupling is reflected in prosody and emphatic stress and may have emerged over evolution, to adapt to gesture-speech biomechanics. In any case, biomechanical constraints might be learned and included in motor control with experience.

Further investigations should be conducted to explore the potential role of these biomechanical processes in communication by constraining speech content and prosodic targets and observing the way participants gesture when reaching these targets. Future studies should also investigate how changes in limb motion, for example, using transitory or more permanent perturbations of the leg may impact speech-motion coupling over time. In particular, investigating variations in speech prosody with adaptation to an increasing effort level of a constrained physical activity could bring more insights into the entanglement between speech and limb movements.

## SUPPLEMENTAL DATA

10.17605/OSF.IO/TSYJK Supplemental Material Fig. S1: https://doi.org/10.17605/OSF.IO/TSYJK;

10.17605/OSF.IO/DETHU OSF Repository Movie: https://doi.org/10.17605/OSF.IO/DETHU;

10.17605/OSF.IO/ZFV75 OSF Repository Scripts: https://doi.org/10.17605/OSF.IO/ZFV75;

10.17605/OSF.IO/K8J6T Scripts and Data sets S1*A*: https://doi.org/10.17605/OSF.IO/K8J6T.

10.17605/OSF.IO/XHK25 Scripts and Data sets S1*B*: https://doi.org/10.17605/OSF.IO/XHK25.

10.17605/OSF.IO/8S5BU Scripts and Data sets S1*C*: https://doi.org/10.17605/OSF.IO/8S5BU.

## GRANTS

This research was jointly supported by the French National Research Agency under Grant ANR-17-FRAL-0005 and the German Research Foundation under Grant DFG FU791/8-1 as part of the SALAMMBO project (Spoken Language in Motions: Learning and Adaptation of Speech Communication in the context of Body Motions).

## DISCLOSURES

No conflicts of interest, financial or otherwise, are declared by the authors.

## AUTHOR CONTRIBUTIONS

H.S., M.D., S.F., and A.R.-C. conceived and designed research; H.S. and S.F. performed experiments; H.S., S.G., and A.R.-C. analyzed data; H.S., M.D., S.F., S.G., and A.R.-C. interpreted results of experiments; H.S. and A.R. prepared figures; H.S. drafted manuscript; H.S., M.D., S.F., and A.R.-C. edited and revised manuscript; H.S., M.D., S.F., and A.R.-C. approved final version of manuscript.
